# Determinants of Cerebral Palsy in Pediatric Patients in Northern Ethiopia: A Hospital-Based Study

**DOI:** 10.1155/2021/9993912

**Published:** 2021-12-20

**Authors:** Peter E. Ekanem, Anne C. K. Nyaga, Niguse Tsegay, Haftamu Ebuy, Elizabeth A. Imbusi, Regina Ekanem, Nissi Peter

**Affiliations:** ^1^Department of Anatomy, College of Health Sciences, Mekelle University, Mekelle, Ethiopia; ^2^Department of Paediatrics and Child Health, College of Health Sciences, Mekelle University, Mekelle, Ethiopia; ^3^Department of Public Health, College of Health Sciences, Mekelle University, Mekelle, Ethiopia

## Abstract

**Introduction:**

Cerebral palsy is the most common neurologic disorder of childhood with lifelong implications in majority of patients. Knowledge of the determinants of cerebral palsy is important for accurate mobilization of resources in obstetric, perinatal, and infant care besides implementation of prevention systems. In Ethiopia, however, this knowledge gap exists as there are no published studies on determinants of cerebral palsy in the country.

**Objective:**

To assess the determinants of cerebral palsy in pediatric patients attending Ayder Comprehensive Specialized Referral Hospital between April 2019 and August 2019.

**Methods:**

An unmatched case-control study was conducted among 50 pediatric cerebral palsy patients and 100 controls, pediatric patients without cerebral palsy or other motor or central nervous system illnesses, attending Ayder Comprehensive Specialized Hospital, Mekelle, Ethiopia. The data were analyzed using SPSS version 27.

**Results:**

Significant factors were operative vaginal delivery (AOR: 9.49, 95% CI: 1.31–68.88), central nervous system infections (AOR: 0.02, 95% CI: 0–0.58), neonatal admissions (AOR: 0.13, 95% CI: 0.03–0.61), and unknown maternal education status (AOR: 18.64, 95% CI: 2.15–161.73).

**Conclusion:**

Operative vaginal delivery, central nervous system infections in infancy, neonatal hospital admissions, and unknown maternal education status were found to be significant determinants for cerebral palsy. This knowledge aids focused hospital and regional health bureau development and implementation of prevention strategies for cerebral palsy, besides improvement of obstetric and neonatal healthcare services, and provides baseline data to the scientific community for further research.

## 1. Introduction

Among the variety of disorders that severely impair motor function in young children, cerebral palsy (CP) is the most prevalent [1]. CP describes a group of permanent disorders considered as nonprogressive disturbances in the developing fetal brain, alterations in fetal development or prematurity complications, or as a result of intrapartum or postnatal insults [2–9].

Population-based studies from around the world report that the prevalence estimates of CP range from 1.5 to more than 4 per 1,000 live births [1, 10–13]. The overall birth prevalence of CP is approximately 2 per 1,000 live births [14–16]. Much of what is known about CP, however, is derived majorly from observations made in high-income countries [17]. This may be because CP studies in sub-Saharan Africa are challenging to conduct, resulting in limited insights into the most basic aspects of CP in Africa including risk factors [18].

Most studies from developed countries portray more prenatal causation than postnatal factors [19], the reverse of which is true in developing countries [20]. Nottidge and Okogbo [21] showed that most CP patients in their study had potentially preventable causes. In a Tanzanian study, CP was primarily related to perinatal problems [22]. A Ghanaian study also demonstrated that severe neonatal hyperbilirubinemia was the most significant and preventable risk for the development of CP [23]. Postnatal causes such as perinatal asphyxia, bilirubin encephalopathy, intracranial infections, ischemic stroke, and congenital brain malformations have been identified as causative factors in other African studies as well [21, 24–26]. Even within resource-limited settings as are found in sub-Saharan Africa, one might anticipate substantial heterogeneity in the occurrence of risk factors for CP [18, 20]. This variability in risk factors necessitates context-specific analyses of CP drivers for the effective management and use of resources.

In a report on CP in Africa involving 22 African countries, Ethiopia was found to have no system for at-risk babies in place for CP [27]. Moreover, a higher prevalence of other conditions in Ethiopia compared with that in other populations has been identified. These include neural tube defects, cleft lip and palate anomalies, rheumatic heart disease, and various anatomical variations [28–31]. Therefore, we sought to investigate what determinant factors were likely to contribute to the development of cerebral palsy in the northern parts of Ethiopia. Moreover, considering that there are no published studies on this condition in Ethiopia, this study aimed at filling this knowledge gap by serving as a benchmark for identification, further investigation, and implementation of preventive systems in the obstetric, neonatal, and infancy period and identification of at-risk babies. This knowledge will also offer context-specific information and data to the policymakers of Ethiopia, to focus available resources in addressing risk factors leading to CP.

## 2. Materials and Methods

### 2.1. Study Setting and Design

This research was conducted in the pediatric outpatient, emergency, and inpatient departments in Ayder Comprehensive Specialized Hospital (ACSH), Mekelle, Ethiopia, from April 2019 to August 2019. Ethiopia's health service is structured into a three-tier system: primary, secondary, and tertiary levels of care. The primary level of care includes primary hospitals, health centers, and health posts. The secondary level of care consists of general hospitals that serve 1 to 1.5 million people, while the tertiary level of health care includes specialized hospitals that serve over 3.5 million people. ACSH is the second largest hospital in Ethiopia, found in Northern Ethiopia's Mekelle City, Tigray Region. It serves a catchment population of over 8 million people, including but not limited to Tigray, Afar, and Southeastern parts of Amhara State.

The study was approved by the Ethics Review Committee of Mekelle University (ERC 1360/2019).

An unmatched case-control study design was used to assess the determinants of cerebral palsy among pediatric patients aged 3 months to 18 years attending ACSH between April 2019 and August 2019. The study population included all pediatric patients with cerebral palsy and control subjects fitting the study criteria attending the hospital's pediatric outpatient, inpatient, and emergency departments during the study period.

### 2.2. Inclusion Criteria for Cases and Controls

Included as cases were all children (1) aged 3 months to 18 years and (2) with a clinical diagnosis of cerebral palsy (motor weakness defined as a score of less than or equal to four of five on the Medical Research Council Scale for Muscle Strength), in at least one limb associated with activity limitation and presumed central origin of weakness based on neurological examination conditional on the caregivers' willingness to participate in the study.

Control subjects were defined as pediatric patients aged less than 18 years recruited from the different pediatric departments at ACSH and free of cerebral palsy or other motor or CNS illnesses. For example, patients with pneumonia and acute gastroenteritis were included as controls.

### 2.3. Exclusion Criteria for Cases and Controls

Pediatric patients who had one or more of the following were excluded from the study in both cases and controls: (1) obstructive hydrocephalus, (2) history of malignancy, (3) evidence of developmental regression not following intracranial infections in the infancy period, (4) diagnosis of a known genetic syndrome, (5) primary neuromuscular disorder, and (6) patients whose caregivers declined to participate in the study.

### 2.4. Sample Size

We identified birth asphyxia, hyperbilirubinemia, and neonatal infections as the main risk factors to calculate the sample size. We chose home delivery as the least important risk factor that would require a large enough sample size to detect the smallest differences in cases and controls. This was chosen based on the likelihood of finding an adequate number of cases in our setting within the period of data collection.

The sample size was calculated using OpenEpi™ version 3.01 open-source calculator function of Sample Size Calculation for Unmatched Case-Control Study [32, 33] at 95% confidence interval (CI) and with the power of 80%, assuming that the proportion of controls with home delivery was 20% and a minimum odds ratio (OR) was 3.26 based on a similar previous study [34]. Considering 1 : 2 ratio of cases to controls, after adding 10% nonresponse rate, the total sample size was 150 (50 cases and 100 controls).

### 2.5. Study Variables

#### 2.5.1. Dependent Variable

The dependent variable was as follows: a clinical diagnosis of cerebral palsy.

#### 2.5.2. Independent Variables

The independent variables were as follows: (1) sociodemographic characteristics (patient's age, sex, residence, and birth order), (2) maternal characteristics (maternal education status), (3) antenatal/pregnancy-related factors (antenatal care (ANC) follow-up, maternal infections, antibiotic use, malaria, use of herbal medications in pregnancy; other complications in pregnancy, e.g., antepartum hemorrhage (APH), preeclampsia and its complications, oligohydramnios, polyhydramnios), (4) intrapartum factors (duration of labor, rupture of membranes, gestational age at birth, place of delivery, mode of delivery, birthweight, Lubchenco classification, multiple delivery, Appearance, Pulse, Grimace, Activity, and Respiration (APGAR) score, time lapse between birth and first cry, resuscitation at birth, nuchal cord, meconium aspiration, birth trauma), and (5) postpartum and infancy factors (congenital anomalies, neonatal admissions, central nervous system CNS infections in infancy, head trauma).

### 2.6. Data Collection Process and Tools

Between April and August 2019, fifteen study staff surveyed the pediatric departments every day for eligible subjects. Caregivers with pediatric patients were approached, and the patients were screened. Eligible patients were invited to participate in the study. Informed verbal or written consent was obtained from each caregiver before the interview. Verbal consent was applied where caregivers were not able to write. Where applicable, the pediatric patient gave verbal assent. To make a diagnosis of CP, caregivers were first interviewed and their responses were confirmed by the evaluation of patients' medical records (where available). This was followed by a physical examination of the child by the senior neurologist or senior resident attached to the pediatric neurology clinic. Two unmatched control subjects were recruited for each case until the sample size was achieved.

Data were collected through caregiver interviews using pretested structured questionnaires. The questionnaire included study variables adapted from reviewing various works of literature on the risk factors associated with the development of CP [26]. All data were verified by the principal investigator before entry into an anonymized database (SPSS).

### 2.7. Data Analysis

The data were cleaned, coded, and analyzed using SPSS software version 27. In the descriptive analysis, we reported frequencies and percentages for all variables. Given the small sample sizes, bivariate analysis was performed using Fisher's exact test and variables significant at an alpha of ≤0.2 were included in the multivariate analysis. Due to the few observations per cell, we used the Firth logistic regression, with the Firth penalized maximum-likelihood estimation method and the Wald method for confidence interval to limit small sample size bias. The multivariate model was built using backward stepwise selection until the remaining variables were significant at alpha ≤0.05. Both crude odds ratio (COR) and adjusted odds ratio (AOR) at 95% confidence interval (95% CI) were reported.

## 3. Results

A total of 50 subjects with cerebral palsy and 100 control subjects were included in the study. The sociodemographic characteristics of the participants are shown in Table 1.

As shown in Table 2, there were no antenatal risk factors significantly associated with CP.

Table 3 shows that intrapartum factors in the univariable analysis significantly associated with CP included the following: mode of delivery (*p*=0.024), birthweight (*p*=0.004), gestational age at birth (*p*=0.030), duration of admission (*p* < 0.001), time to cry after birth (<0.001), and Lubchenco class (*p*=0.005). Significant postnatal factors included neonatal illness without admission (*p*=0.108), neonatal admissions (*p* < 0.001), CNS infection in infancy (*p* < 0.001), and immunization as per the Expanded Program on Immunization (EPI) schedule (*p*=0.070).

In the fully adjusted model (Table 4), operative vacuum-assisted delivery (AOR: 9.49, 95% CI: 1.31–68.88), unknown maternal education status (AOR: 18.64, 95% CI: 2.15–161.73), CNS infection in infancy (AOR: 0.02, 95% CI: 0–0.58), and neonatal admission (AOR: 0.13, 95% CI: 0.03–0.61) remained statistically significant.

**Figure 1:**
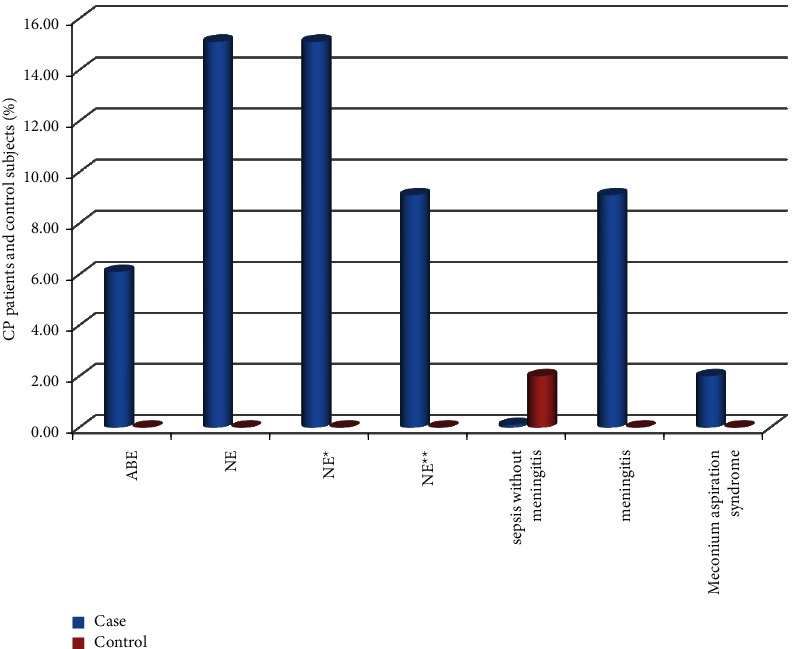
Neonatal admission diagnoses among CP patients and control subjects, ACSH (April-August, 2019). NE: neonatal encephalopathy; NE ^*∗*^: stage II neonatal encephalopathy. NE ^*∗∗*^: stage III neonatal encephalopathy; ABE: acute bilirubin encephalopathy; MAS: meconium aspiration syndrome.

As shown in Figure 1, diagnoses in the study participants who had been admitted in the neonatal period with documentation available were mostly found only among the cases and not the controls except in the case of neonatal sepsis. The most common diagnoses were as follows: unspecified neonatal encephalopathy (15.10% of cases), stage II neonatal encephalopathy (15.1% of cases), stage III neonatal encephalopathy (9.10% of cases), and meningitis (9.10% of cases).

## 4. Discussion

In this study, a male predisposition was found among patients with cerebral palsy with a male: female ratio of 1.3 : 1. This is congruent with other studies that revealed a male predisposition for CP with a ratio of 1.3 to 1.4 : 1 [35, 36]. A biological vulnerability for the male sex has been suggested to be due to possible differences in brain organization, genetic disorders, or the influence of female hormones with a possible reduction in the effects of brain damage [36–38]. Most of the patients with CP also had higher birth order. This is consistent with another study carried out in India by Sharma et al. [39], which showed a higher prevalence of CP in patients with higher birth order but was at variance with the findings of MacLennan et al. [2], who found in their study that patients with CP were more among lower birth order patients. The implication of our finding could be related to younger mothers, who have higher-order birth without adequate knowledge of pregnancy-related complications, hence resulting in delayed health-seeking behavior compared to mothers with higher parity.

Operative vaginal deliveries (vaginal delivery accomplished with the aid of instruments, which can be vacuum or forceps) [40] were found to be significantly associated with the development of CP in our study. All such patients had been delivered through vacuum-assisted delivery. Instrumental deliveries (compared with spontaneous vaginal or elective cesarean deliveries) were associated with an increased risk of CP in a systematic review of risk factors for cerebral palsy in children born at term in developed countries [41]. Operative delivery was, however, not reported as a common etiology among African countries in a separate study [20]. Our study seems to concur with the widely believed fact that vacuum delivery is associated with an increased risk of traumatic and nontraumatic intracranial hemorrhages and a higher risk of convulsions or encephalopathy as compared with infants delivered vaginally without operative assistance [42].

We also found CNS infections in infancy to be determinant for CP. This is comparable with a Nigerian study by Eyong et al. [43] in which a history of CNS infection was found in about a third of the cases. Similar findings have also been found in Uganda and Botswana [24, 25]. A different view was found in a systematic review of CP in Africa [20], which showed that the most commonly reported etiologies identified in African cohorts were in the perinatal period. Some studies have shown that a significant number of children that had meningitis developed moderate-to-severe neurodevelopmental impairment and that poorly treated meningitis could result in brain damage leading to long-term neurologic sequelae [44, 45]. A similar explanation could be implicated in our findings.

Moreover, CP was associated with a history of hospital admission in the neonatal period. Of the patients who had documentation of diagnoses during neonatal admission, the majority had perinatal asphyxia with features of neonatal encephalopathy, neonatal infections, and acute bilirubin encephalopathy. Patients with CP also had longer durations of admission compared with the controls. These diagnoses, which were some of the reasons for admission in our study, have also been identified in other studies in Nigeria and India as risk factors for CP [46, 47] and are often associated with longer durations of treatment [48]. This association may explain the predisposition of patients admitted in the newborn period to developing CP as observed in our study.

Unknown maternal education status was found in our study to be significantly associated with cerebral palsy. More than half of the patients who had unknown maternal education had also been admitted in the neonatal period and among them also were patients who had had CNS infections in infancy. These factors that were also found to be determinant for CP in our study may be the reason for this significance.

### 4.1. Limitations

This study relied on the ability of parents or guardians to recall events that happened in the past in assessing risk factors. This could have led to recall bias and therefore an over- or underestimation of the risk factors, which may also have been enhanced by the age difference between the cases and the controls. Caregivers, such as relatives who accompanied some of the participants, did not know some of the maternal or infant's characteristics, thus giving rise to the “unknowns,” which could have affected or influenced the study outcomes. The study also had a small sample size, which might not have been large enough to detect some differences in the risk factors. Additionally, although the CP patients included were those assessed and diagnosed in the pediatric neurology clinic, genetic testing, which is not readily available in our setup, was not done in patients without known genetic syndromes to preclude unknown syndromes and other genetic diseases.

## 5. Conclusion and Recommendation

From these study findings, the development of CP in our setup is associated with operative vacuum-assisted delivery, history of CNS infections in infancy, and the need for hospital admission in the neonatal period.

Operative vaginal delivery has not been found to be a significant risk factor for CP in other African studies. We recommend that further study be done by Ayder Hospital and the Regional Health Bureau in conjunction with the Ministry of Health to evaluate what factors predispose babies born through vacuum-assisted delivery to CP in both Ayder Hospital and the various health facilities in the hospital's catchment area.

We also recommend that the Regional Health Bureau and the Ministry of Health carry out further research in the region's health facilities on the risk factors that predispose to neonatal encephalopathy and CNS infections in infancy and systems be put in place to mitigate avoidable causes.

## Figures and Tables

**Table 1:** Sociodemographic characteristics among pediatric CP patients and control subjects in ACSH (April-August 2019).

		Cases *N* = 50	Controls *N* = 100	*p* value
		*n* (%)	*n* (%)	
Age	<5 years	36 (72.0)	53 (53.0)	**0.034**
	≥5 years	14 (28.0)	47 (47.0)	

Sex	Male	29 (58.0)	57 (57.0)	0.315
	Female	21 (42.0)	38 (38.0)	
	ND	0 (0.0)	5 (5.0)	

Residence	Rural	16 (32.7)	41 (41.0)	0.372
	Urban	33 (66.3)	59 (59.0)	
	ND	1 (1.0)	0 (0.0)	

Birth order	<5th child	43 (86.0)	87 (87.9)	0.797
	≥5th child	7 (14.0)	12 (12.1)	
	ND	0 (0)	1 (1.0)	

Maternal education	None	8 (21.1)	30 (78.9)	**<0.001**
	Primary	6 (20)	24 (80)	
	Secondary	20 (44.4)	25 (55.6)	
	Tertiary	2 (9.1)	20 (90.9)	

Maternal age	<25 years	14 (29.8)	28 (28.9)	0.657
	25–29 years	17 (36.2)	28 (28.9)	
	30–34 years	7 (14.9)	24 (24.7)	
	35–39 years	8 (17.0)	13 (13.4)	
	>40 years	1 (2.1)	4 (4.1)	

CP: cerebral palsy; ND: not documented.

**Table 2:** Antenatal factors among CP patients and control subjects in ACSH (April-August 2019).

Variables		Cases (50) *N* (%)	Controls (100) *N* (%)	*p* value
Maternal fever in pregnancy	Yes	0 (0.0)	4 (4.0)	0.291
	No	50 (100.0)	93 (93.9)	
	Unknown	0 (0.0)	3 (2.0)	

Maternal antibiotic use in pregnancy	Yes	1 (2.0)	5 (5.1)	0.459
	No	49 (98.0)	91 (91.9)	
	Unknown	0 (0.0)	3 (3.0)	

ANC	Yes	48 (96.0)	96 (96.0)	>0.999
	No	2 (4.0)	3 (3.0)	
	Unknown	0 (0.0)	1 (1.0)	

ANC visits	Below 4 visits	16 (34.0)	21 (22.3)	0.320
	At least 4 visits	31 (66.0)	73 (77.7)	
	Unknown	3 (6.0)	6 (6.0)	

Malaria in pregnancy	Yes	0 (0.0)	4 (4.0)	0.290
	No	50 (100.0)	94 (94.0)	
	Unknown	0 (0.0)	2 (2.0)	

Other maternal complications in pregnancy	Yes	3 (6.0)	6 (6.0)	0.874
	No	47 (94.0)	92 (92.0)	
	Unknown	0 (0.0)	2 (2.0)	

Type of pregnancy complication	GDM	0 (0.0)	1 (1.0)	0.786
	Preeclampsia	1 (2.0)	3 (3.0)	
	APH	1 (2.0)	2 (2.0)	
	Syphilis	1 (2.0)	0 (0.0)	
	Unknown	47 (96.0)	94 (94.0	

Maternal chronic illness	Yes	0 (0.0)	2 (2.0)	0.553
	No	50 (100.0)	98 (98.0)	

Type of chronic illness	DM	0 (0.0)	0 (0.0)	NA
	Syphilis	0 (0.0)	0 (0.0)	
	Hypertension	0 (0.0)	3 (3.0)	
	HIV	0 (0.0)	0 (0.0)	
	Cardiac	0 (0.0)	0 (0.0)	
	Asthma	0 (0.0)	0 (0.0)	
	Unknown	50 (100.0)	97 (97.0)	

Use of herbal medicine during pregnancy	Yes	0 (0.0)	0 (0.0)	0.553
	No	50 (100.0)	98 (98.0)	
	Unknown	0 (0.0)	2 (2.0)	

Use of fertility intervention	Yes	0 (0.0)	4 (4.0)	0.302
	No	50 (0.0)	96 (96.0)	

Consanguinity	Yes	0 (0.0)	1 (1.0)	>0.999
	No	50 (100.0)	99 (99.0)	

CP: cerebral palsy; ACSH: Ayder Comprehensive Specialized Hospital; ANC: antenatal care; GDM: gestational diabetes mellitus; APH: antepartum hemorrhage; DM: diabetes mellitus; HIV: human immunodeficiency virus; APGAR: Appearance, Pulse, Grimace, Activity, and Respiration; CNS: central nervous system; EPI: Expanded Program on Immunization.

**Table 3:** Intrapartum and postpartum factors among CP patients and control subjects in ACSH (April-August 2019).

Variables		Cases (50) *N* (%)	Controls (100) *N* (%)	*p* value
Pregnancy outcome	Singleton	49 (98.0)	97 (97.0)	>0.999
	Multiple	1 (2.0)	3 (3.0)	

Rupture of membranes	<18 hr	26 (52.0)	58 (59.8)	0.385
	≥18 hr	3 (6.0)	2 (2.1)	
	Unknown	21 (42.0)	37 (38.1)	

Place of delivery	Home	4 (8.9)	14 (14.1)	0.634
	Health center	12 (26.7)	21 (21.2)	
	Primary hospital	8 (17.8)	21 (21.2)	
	Referral hospital	8 (17.8)	23 (23.2)	
	General hospital	13 (28.9)	20 (20.2)	

Mode of delivery	SVD	40 (80.0)	89 (89.0)	**0.024**
	Cesarean section	3 (6.0)	9 (9.0)	
	Assisted breech	1 (2.0)	0 (0.0)	
	Instrumental vacuum	6 (12.0)	2 (2.0)	

Birth trauma	Yes	3 (6.0)	2 (2.0)	0.534
	No	46 (92.0)	95 (95.0)	
	Unknown	1 (2.0)	3 (3.0)	

Congenital anomaly	Yes	0 (0)	6 (6.0)	**0.179**
	No	50 (100)	94 (94.0)	

Birthweight	<1.5 kg	0 (0.0)	0 (0.0)	**0.004**
	1.5–2.499 kg	8 (16.0)	1 (1.0)	
	2.5–3.9 kg	23 (46.0)	52 (52.0)	
	>=4 kg	1 (2.0)	6 (6.0)	
	Unknown	18 (36.0)	41 (41.0)	

Gestational age at birth	Preterm	7 (14.3)	3 (3.1)	**0.030**
	Term	41 (83.7)	92 (95.8)	
	Postterm	1 (2.0)	1 (1.0)	

Duration of labor	Normal	6 (12.0)	13 (13.0)	0.903
	Shorter	14 (28.0)	29 (29.0)	
	Longer	13 (26.0)	30 (30.0)	
	Unknown	17 (34.0)	28 (28.0)	

Duration of admission	<=7 days	7 (14.0)	4 (4.0)	**<0.001**	
	>7 days	25 (50.0)	3 (3.0)	
	Unknown	18 (36.0)	93 (93.0)	

Parity	First pregnancy	5 (10.0)	6 (6.0)	0.672
	At least 2nd pregnancy	45 (90.0)	94 (94.0)	

Infant cried immediately after birth	Yes	28 (56.0)	97 (97.0)	**<0.001**
	No	22 (44.0)	3 (3.0)	

Lubchenco class	SGA	6 (12.0)	0 (0.0)	**0.005**
	AGA	22 (44.0)	53 (54.1)	
	LGA	2 (4.0)	7 (7.1)	
	Unknown	20 (40.0)	38 (38.8)	

Neonatal illness without admission	Yes	3 (6)	1 (1)	**0.108**
	No	47 (94)	99 (99)	

Neonatal admission	Yes	33 (66.0)	7 (7.0)	**<0.001**
	No	17 (34.0)	93 (93.0)	

Birth trauma	Yes	3 (6.0)	2 (2.0)	0.543
	No	46 (92.0)	95 (95.0)	
	Unknown	1 (2.0)	3 (3.0)	

CNS infection in infancy	Yes	8 (16.3)	0 (0.0)	**<0.001**
	No	41 (83.7)	100 (100)	

EPI immunization	Yes	46 (92.0)	98 (98.0)	**0.070**
	No	1 (2)	2 (2)	
	Unknown	3 (6)	0 (0.0)	

CP: cerebral palsy; ACSH: Ayder Comprehensive Specialized Hospital; CNS: central nervous system; EPI: Expanded Program on Immunization; SGA: small for gestational age; AGA: appropriate for gestational age; LGA: large for gestational age; SVD: spontaneous vertex delivery. Bold values indicate variables found significant in the univariate analysis at an alpha of 0.2 and included in the multivariate analysis.

**Table 4:** Risk factors for CP among pediatric patients attending ACSH (April-August 2019).

		COR (95% CI) *p* value	AOR (95% CI) *p* value
Patient age	<5 years	1	
	≥5 years	0.45 (0.22–0.93) 0.03	

Maternal education	None	1	1
	Primary	0.95 (0.3–3.06) 0.934	0.73 (0.16–3.36) 0.691
	Secondary	3.03 (1.15–7.93) 0.024	1.14 (0.31–4.21) 0.843
	Tertiary	0.44 (0.09–2.06) 0.295	0.59 (0.1–3.61) 0.567
	Unknown	32.29 (4.79–217.73) <0.001	18.64 (2.15–161.73) **0.008**

Mode of delivery	SVD	1	1
	Cesarean section	0.81 (0.22–3.07) 0.762	0.42 (0.02–8.54) 0.571
	Assisted breech	6.63 (0.07–622.16) 0.414	31.48 (0.29–3440.91) 0.15
	Instrumental vacuum	5.75 (1.17–28.21) 0.031	9.49 (1.31–68.88) **0.026**

Congenital anomaly	Yes	1	
	No	6.95 (0.3–158.24) 0.224	

Birthweight	1.5–2.499 kg	1	
	2.5–3.9 kg	0.08 (0.01–0.53) 0.009	
	>=4 kg	0.04 (0–0.57) 0.017	
	Unknown	0.08 (0.01–0.53) 0.009	

Gestational age at birth	Preterm	1	
	Term	0.21 (0.05–0.83) 0.026	
	Postterm	0.47 (0.02–10.1) 0.627	

Duration of admission	≤7 days	1	1
	>7 days	4.37 (0.82–23.18) 0.083	3.75 (0.61–23.11) 0.154
	Not admitted	0.12 (0.03–0.44) 0.002	0.13 (0.03–0.61) **0.009**

Infant cried immediately after birth	Yes	1	
	No	21.99 (6.48–74.68) <0.001	

Lubchenco classification	SGA	1	
	AGA	0.03 (0–0.75) 0.033	
	LGA	0.03 (0–0.81) 0.038	
	Unknown	0.04 (0–0.96) 0.047	

Neonatal illness without admission	Yes	1	
	No	0.2 (0.02–1.79) 0.151	

Neonatal admission	Yes	1	
	No	0.04 (0.02–0.11) <0.001	

CNS infection in infancy	Yes	1	1
	No	0.02 (0–0.51) 0.017	0.02 (0–0.58) **0.023**

EPI immunization	Yes	1	
	No	1.27 (0.12–13.51) 0.842	
	Unknown	14.83 (0.48–462.18) 0.124	

CP: cerebral palsy; ACSH: Ayder Comprehensive Specialized Hospital; CNS: central nervous system; EPI: Expanded Program on Immunization; SGA: small for gestational age; AGA: appropriate for gestational age; LGA: large for gestational age; SVD: spontaneous vertex delivery.

## Data Availability

The data used to support the findings of this study are included within the article.

## References

[1] Paneth N., Hong T., Korzeniewski S. (2006). The descriptive epidemiology of cerebral palsy. *Clinics in Perinatology*.

[2] MacLennan A. H., Thompson S. C., Gecz J. (2015). Cerebral palsy: causes, pathways, and the role of genetic variants. *American Journal of Obstetrics and Gynecology*.

[3] Trønnes H., Wilcox A. J., Lie R. T., Markestad T., Moster D. (2014). Pathologic intrauterine processes risk of cerebral palsy in relation to pregnancy disorders and preterm birth: a national cohort study. *Developmental Medicine and Child Neurology*.

[4] Grobman W. A., Lai Y., Rouse D. J., Spong C. Y., Varner M. W., Mercer B. M. (2013). The association of cerebral palsy and death with small-for-gestational-age birthweight in preterm neonates by individualized and population-based percentiles. *American Journal of Obstetrics and Gynecology*.

[5] Blair E. M., Nelson K. B. (2015). Fetal growth restriction and risk of cerebral palsy in singletons born after at least 35 weeks’ gestation. *American Journal of Obstetrics and Gynecology*.

[6] Mor O., Stavsky M., Yitshak-Sade M. (2016). Early onset preeclampsia and cerebral palsy: a double hit model?. *American Journal of Obstetrics and Gynecology*.

[7] Gomez R., Romero R., Ghezzi F., Yoon B. H., Mazor M., Berry S. M. (1998). The fetal inflammatory response syndrome. *American Journal of Obstetrics and Gynecology*.

[8] Yoon B. H., Park C.-W., Chaiworapongsa T. (2003). Intrauterine infection and the development of cerebral palsy. *BJOG: An International Journal of Obstetrics and Gynaecology*.

[9] Moshe S., Omer M., Salvatore A. M., Shirley G., Nandor Gt E. O. (2017). Cerebral palsy-trends in epidemiology and recent development in prenatal mechanisms of disease, treatment, and prevention. *Frontiers in Pediatrics*.

[10] Mutch L., Alberman E., Hagberg B., Kodama K., Perat M. V. (2008). Cerebral palsy epidemiology: where are we now and where are we going?. *Developmental Medicine and Child Neurology*.

[11] Arneson C. L., Durkin M. S., Benedict R. E. (2009). Prevalence of cerebral palsy: autism and developmental disabilities monitoring network, three sites, United States, 2004. *Disability and Health Journal*.

[12] Bhasin T. K., Brocksen S., Avchen R. N., Van Naarden Braun K, Braun K. (2006). Prevalence of four developmental disabilities among children aged 8 years-metropolitan atlanta developmental disabilities surveillance program, 1996 and 2000. *Morbidity and Mortality Weekly Report Surveillance Summaries*.

[13] Johnson A. (2002). Prevalence and characteristics of children with cerebral palsy in Europe. *Developmental Medicine and Child Neurology*.

[14] Winter S., Autry A., Boyle C., Yeargin-Allsopp M. (2002). Trends in the prevalence of cerebral palsy in a population-based study. *Pediatrics*.

[15] Odding E., Roebroeck M. E., Stam H. J. (2006). The epidemiology of cerebral palsy: incidence, impairments and risk factors. *Disability & Rehabilitation*.

[16] Hirtz D., Thurman D. J., Gwinn-Hardy K., Mohamed M., Chaudhuri A. R., Zalutsky R. (2007). How common are the “common” neurologic disorders?. *Neurology*.

[17] Dan B., Paneth N. (2017). Making sense of cerebral palsy prevalence in low-income countries. *The Lancet*.

[18] Birbeck G. L. (2018). The burden of cerebral palsy in africa-new insight reveal more epidemiological complexity.

[19] Paneth N., Leviton A., Goldstein M. (2007). A report: the definition and classification of cerebral palsy. *Developmental Medicine & Child Neurology-Supplement*.

[20] Donald K. A., Samia P., Kakooza-Mwesige A., Bearden D. (2014). Pediatric cerebral palsy in Africa: a systematic review. *Seminars in Pediatric Neurology*.

[21] Nottidge V. A., Okogbo M. E. (1991). Cerebral palsy in ibadan, Nigeria. *Developmental Medicine and Child Neurology*.

[22] Kisanga A. O., Verma A., Bhaskaran A. A., Elangovan M. (2012). Prevalence of cerebral palsy in children under five in and around dar-Es-salaam. *IMTU Medical Journal*.

[23] Atiemo E., Onike R., Badoe E. (2015). Classification and risk factors for cerebral palsy in the korle bu teaching hospital, accra: a case-control study. *Paediatrics*.

[24] Kakooza-Mwesige A., Andrews C., Peterson S., Mangen F. W., Eliasson A. C., Forssberg H. (2017). Prevalence of cerebral palsy in Uganda: a population-based study. *Lancet Glob Health*.

[25] Monokwane B., Johnson A., Gambrah-Sampaney C. (2017). Risk factors for cerebral palsy in children in Botswana. *Pediatric Neurology*.

[26] Bearden D. R., Monokwane B., Khurana E. (2016). Pediatric cerebral palsy in Botswana: etiology, outcomes, and comorbidities. *Pediatric Neurology*.

[27] Donald K. A., Kakooza A. M., Wammanda R. D. (2015). Pediatric cerebral palsy in Africa. *Journal of Child Neurology*.

[28] Bekele K. K., Ekanem P. E., Meberate B. (2019). Anatomical patterns of cleft lip and palate deformities among neonates in Mekelle, Tigray, Ethiopia; implication of environmental impact. *BMC Pediatrics*.

[29] Berihu B. A., Welderufael A. L., Berhe Y. (2018). High burden of neural tube defects in Tigray, Northern Ethiopia: hospital-based study. *PLoS One*.

[30] Ekanem P. E., Abba S., Abba S., Mariam K., Assefa H. (2015). Variations of sciatic nerve bifurcation in dissected cadaveres from Ethiopia and their clinical implication: a case report. *International Journal of Anatomy and Research*.

[31] Yadeta D., Hailu A., Haileamlak A. (2016). Prevalence of rheumatic heart disease among school children in Ethiopia: a multisite echocardiography-based screening. *International Journal of Cardiology*.

[32] Kelsey J. L., Whittemore A. S., Evans A. S., Thompson W. D. (1996). *Methods in Observational Epidemiology*.

[33] Fleiss J. L. (1981). *Statistical Methods for Rates and Proportions*.

[34] Ejeliogu E., Ebonyi A., John C., Yiltok E., Toma B. (2017). An evaluation of risk factors for cerebral palsy in children in jos, Nigeria. *British Journal of Medicine and Medical Research*.

[35] Himmelmann K., Uvebrant P. (2014). The panorama of cerebral palsy in Sweden. XI. Changing patterns in the birth-year period 2003-2006. *Acta Paediatrica*.

[36] Stanley F., Blair E., Alberman E. (2000). *Cerebral Palsies: Epidemiology and Causal Pathways*.

[37] Reiss A. L., Kesler S. R., Vohr B. (2004). Sex differences in cerebral volumes of 8-year-olds born preterm. *The Journal of Pediatrics*.

[38] Vasileiadis G. T., Thompson R. T., Han V. K. M., Gelman N. (2009). Females follow a more “compact” early human brain development model than males. a case-control study of preterm neonates. *Pediatric Research*.

[39] Sharma P., Sharma U., Kabra A. (1999). Cerebral palsy-clinical profile and predisposing factors. *Indian Pediatrics*.

[40] Unzila A., Errol R. (2009). Vacuum-assisted vaginal delivery. *Reviews in Obstetrics and Gynecology*.

[41] McIntyre S., Blair E., Badawi N., Keogh J., Nelson K. B. (2013). Antecedents of cerebral palsy and perinatal death in term and late preterm singletons. *Obstetrics & Gynecology*.

[42] Ekéus C., Högberg U., Norman M. (2014). Vacuum assisted birth and risk for cerebral complications in term newborn infants: a population-based cohort study. *BMC Pregnancy and Childbirth*.

[43] Eyong K. I., Ekanem E., Asindi A. (2017). Challenges of care givers of children with cerebral palsy in a developing country. *International Journal of Contemporary Pediatrics*.

[44] Seale A. C., Blencowe H., Blencowe H. (2013). Neonatal severe bacterial infection impairment estimates in South Asia, sub-Saharan Africa, and Latin America for 2010. *Pediatric Research*.

[45] Edmond K., Clark A., Korczak V. S., Sanderson C., Griffiths U. K., Rudan I. (2010). Global and regional risk of disabling sequelae from bacterial meningitis: a systematic review and meta-analysis. *The Lancet Infectious Diseases*.

[46] Adogu P., Ubajaka C., Egenti N., Obinwa A., Igwe W. (2016). Evaluation of risk factors of cerebral palsy in a tertiary health facility, Nnewi, Nigeria: a case-control study. *International Journal of Medical Science and Public Health*.

[47] Jain V., Jain J., Singh G., Pandey A. (2015). Perinatal risk factors in cerebral palsy: a rehab center based study. *Indian Journal of Cerebral Palsy*.

[48] Kliegman R., Stanton B., St Geme J., Schor N. F., Behrman R. E. (2019). *Nelson Textbook of Paediatrics*.

